# A twin study of cilioretinal arteries, tilted discs and situs inversus

**DOI:** 10.1007/s00417-017-3859-7

**Published:** 2017-12-14

**Authors:** Alex J. Baneke, Katie M. Williams, Omar A. Mahroo, Moin Mohamed, Christopher J. Hammond

**Affiliations:** 1 Department of Ophthalmology, King’s College London, St Thomas’ Hospital Campus, Westminster Bridge Road, London, SE1 7EH UK; 2 Department of Ophthalmology, St Thomas’ Hospital, Westminster Bridge Road, London, SE1 7EH UK; 3 Department of Twin Research and Genetic Epidemiology, King’s College London, St Thomas’ Hospital Campus, Westminster Bridge Road, London, SE1 7EH UK; 4 Moorfields Eye Hospital, 162 City Rd, London, EC1V 2PD UK

**Keywords:** Cilioretinal arteries, Heritability, Twin, Situs inversus, Tilted disc

## Abstract

**Purpose:**

To establish the prevalence and heritability of cilioretinal arteries (CRAs), tilted discs (TDs) and situs inversus (SI).

**Methods:**

Fundus photos from the Twins UK Adult Twin registry twin database were analyzed: 1812 individuals, 526 complete monozygotic (MZ) twin pairs and 336 complete dizygotic (DZ) pairs. Images were assessed non-stereoscopically on a computer screen by the same ophthalmologist for presence of CRAs, TDs or SI. Prevalence figures, probandwise concordances and heritabilities were calculated.

**Results:**

Prevalence of a CRA in subjects’ right eyes was 28.6% (26.5–30.8). Prevalence of subjects with a CRA in at least one eye was 45.0% (42.6–47.5), with a TD in at least one eye was 1.2% (0.8–1.9), and with SI at least one eye was 0.5% (0.3–1.0). There was no association between birth weight and presence of CRA.

Concordance for CRA in at least one eye (MZ twins) was 60% (95% CI 55–64), and (DZ) was 45% (95% CI 39–51). Heritability for CRAs in at least one eye was 49.4% (95% CI 38.1–59.7) and for both eyes was 32.9% (95% CI 10.4–53.3). We were unable to calculate meaningful heritabilities or concordances for TDs and situs SI, due to insufficient numbers.

**Conclusions:**

The presence of CRAs appears to be moderately heritable, with greater variance explained by individual environmental factors or even stochastic events. They were not associated with low birth weight. Future genetic research and studies of birth/lifecourse cohorts may offer further insights into the etiology of congenital papillovascular abnormalities.

**Electronic supplementary material:**

The online version of this article (10.1007/s00417-017-3859-7) contains supplementary material, which is available to authorized users.

## Introduction

The morphology of the optic disc and its associated vasculature is clinically important as it plays a role in a number of ocular diseases, including anterior ischaemic optic neuropathy, central retinal artery occlusion and the tilted disc syndrome. While genetic factors underlying optic disc size are being discovered [1, 2], little is known about which factors influence the development of cilioretinal arteries, tilted discs and situs inversus, and the extent to which they are heritable.

In around two thirds of eyes, the inner retina is supplied by the central retinal artery only. However, in a third of eyes a cilioretinal artery branches off from the short posterior ciliary arteries to supply part of the inner retina [3–5]. The presence of a cilioretinal artery can influence the outcome of several retinal vascular pathologies [6–8] In cases of central retinal artery occlusion, areas of the retina supplied by a cilioretinal artery still receive a blood supply and, therefore, the corresponding visual field is preserved [7]. Equally, temporal cilioretinal arteries may provide an increased blood supply to the temporal side of the optic disc and, therefore, preserve visual field and acuity in advanced open angle glaucoma [6]. Conversely, it has been suggested that the presence of a cilioretinal artery may increase the risk of diabetic macular oedema [8].

The population prevalence of cilioretinal arteries has been reported in between 18 and 32% of eyes and 35–49% of individuals [3, 4]. Differences may have resulted from methods (use of fluorescein angiography or not), or ethnic differences within the populations studied. A previous small study examined cilioretinal artery heritability (in 112 twin pairs) and found significant evidence of a genetic effect underlying the presence of cilioretinal arteries, with a heritability of 74% (95% CI: 34–94%) for cilioretinal arteries in both eyes [5], albeit with wide confidence intervals. Little is known about environmental factors that may affect the development of cilioretinal arteries, though birth weight may play a role [9]. Varying oxygen levels has been shown to play a role in the development of retinal vasculature in mice [10].

Tilted optic discs occur in 1–2% of the population and their hereditary pattern has not been assessed [11–14]. Classically, the inferior portion of the optic disc has a crescentic shape, one side of the disc is depressed, and the blood vessels enter the eye obliquely [15]. They are one aspect of the tilted disc syndrome, which is composed of a number of signs: astigmatic refractive error, superotemporal visual field loss, situs inversus of the retinal blood vessels, β-peripapillary atrophy, inferonasal chorioretinal thinning and posterior staphyloma or coloboma. This syndrome is thought to be caused by incomplete closure of the embryonic fetal fissure with the resulting formation of a coloboma at the inferonasal aspect of the disc [11]. This congenital syndrome is in contrast to acquired myopic tilted discs (not part of the tilted disc syndrome), which predominantly occur temporally. The tilted disc syndrome may be familial, and an autosomal dominant pattern of inheritance has been suggested in one small series of three patients with the tilted disc syndrome and lacquer cracks within the same family [16–18].

Situs inversus of the optic disc is a characteristic emergence of the retinal vessels in an anomalous, nasal direction, followed by an abrupt turn towards the temporal side, associated with dysversion of the optic nerve head [19]. It is thought to be a congenital anomaly and also occurs in tilted disc syndrome. A study of 4324 subjects found situs inversus in 0.21% [19].

The aim of this study was to identify the heritability of cilioretinal arteries, tilted discs, and situs inversus in a large twin population.

## Methods

We examined color fundus photographs taken from the TwinsUK Adult Twin registry, held at King’s College, London, UK [20]. This registry has been compiled from the general population through national media campaigns, and comprises over 12,000 predominantly female Caucasian ancestry twins (in part from initial recruitment of women, and subsequently a female volunteer bias common to twin registries), from throughout the United Kingdom. Twins involved have been shown to be comparable to women from the age-matched general population for a wide number of medical traits [21]. Twins gave fully informed consent under a protocol reviewed by the St. Thomas’s Hospital Local Research Ethics Committee (EC04/015), which was performed in accordance with the Helsinki Declaration. Zygosity had been determined from standardized questionnaires and confirmed with genome wide analyses.

Images were available for 1812 individuals, [1101 monozygotic (MZ) and 711 dizygotic (DZ) twins]. Of these, there were 526 complete MZ twin pairs and 336 complete DZ pairs. Some images were of insufficient quality for grading: the total numbers of images graded were 1665 right eyes and 1634 left eyes. Individuals were recruited from the United Kingdom and were predominantly Caucasian. Subjects were predominantly female: 1796 were female and 16 male. Images were taken with two cameras as the database was updated over time. Cameras used were: the Nidek model 3-DX stereo camera, Gamagori, Japan (film: Polaroid, Minnetonka, MN, USA) and the Nidek digital nonmydriatic fundus camera, model AFC210, Japan.

All images were assessed in a 2D format on a computer screen by the same ophthalmologist (AB), and a subset of 279 subjects were graded again by a senior ophthalmologist (KW) to assess levels of agreement. The performance of the two graders was compared using the kappa statistic. A cilioretinal artery was defined as an artery non-contiguous with the central retinal artery, emerging at the edge of the optic disc and typically exhibiting a 180 ° turn to supply the retina. Images were graded for presence of cilioretinal artery and position by quadrant of the optic disc (superior nasal, inferior nasal, superior temporal and inferior temporal). Situs inversus of the optic disc was defined as an emergence of the retinal vessels in an anomalous nasal direction followed by a sharp turn towards the temporal retina, associated with dysversion of the optic nerve head [19]. A tilted disc was defined as a tilted appearance of the disc, with a ratio of minimum to maximum optic disc diameter of less than 0.75 [22]. All images of situs inversus and tilted discs were confirmed by an experienced medical retina specialist (MM).

To examine the impact of birthweight on cilioretinal artery prevalence, we compared the prevalence between groups stratified by birthweight.

## Statistics

The groups of MZ and DZ twins were compared using chi-squared and two sided t-tests for any differences in age, sex and prevalence of gradable eyes containing a cilioretinal artery. Figures for prevalence were calculated as a proportion of the total number of gradable images.

Probandwise concordances were calculated using the formula:

$$ \mathrm{probandwise}\  \mathrm{concordance}=2\mathrm{C}/\left(2\mathrm{C}+\mathrm{D}\right) $$

, where C = the number of twin pairs where both twins have the particular optic disc morphology being analyzed, and D = the number of twin pairs where one has the particular optic disc morphology and the other does not. Probandwise concordance rates estimate the risk of a twin having a particular optic disc morphology, given the fact that their twin has that optic disc morphology. Microsoft Excel and Stata (Stata statistical software version 13.1, StataCorpLP, College Station, TX, USA) were used for data handling and analysis.

Heritability was calculated using maximum likelihood structural equation twin modeling using the OpenMx package (http://openmx.psyc.virginia.edu) in R (http://www.r-project.org). In twin modeling the phenotypic variance of a trait is partitioned into the additive genetic effects (A), non-additive genetic effects (D), the shared environment between siblings (C), and the individual specific environment effects (E). Threshold liability ACE/ADE models were constructed for each trait with standardized path coefficients and expected variance and covariance matrices. The goodness of fit of the full and reduced models, with parameters removed in a step-wise manner, is compared to the observed data using χ^2^ tests. The most parsimonious, best-fitting model to explain the observed data is selected by identifying that with the minimum Akaike’s information criterion (AIC). Heritability is calculated as the proportion of total trait variance attributable to the additive genetic effect (A) +/− the non-additive genetic effect in the best fitting model. A binary grading of presence or absence of congenital papillovascular morpholgies was used for all traits.

## Results

Including incomplete twin pairs, 1665 right eyes and 1634 left eyes were gradable; 1530 subjects had both eyes gradable. Of complete twin pairs, there were 1014 gradable MZ right eyes and 989 left eyes, 651 gradable DZ right eyes and 645 left eyes. 887 MZ twins and 643 DZ twins had both eyes gradable. Data on birth weight was available for 763 twins.

When comparing baseline characteristics of MZ and DZ twin groups (Table 1), MZ twins had more male subjects, but the overall number of men was low in both groups (14 in MZ and 2 in DZ). The mean age of the MZ twins was significantly lower than the DZ group: MZ = 56.6 (10.5), DZ = 58.4 (9.5), *p* = 0.0003.

**Table 1:** Characteristics of overall study participants (complete pairs)

Characteristic	MZ twins	DZ twins	*p*-value
Subjects, including incomplete pairs	1101	711	
Complete pairs	526	336	
Gradable eyes, including incomplete pairs (RE/LE)	1014/989	651/645	0.83 (chi-squared)
Male/Female, including incomplete pairs	14/1087	2/709	0.03 (chi-squared)
Age in years, including incomplete pairs (average and SD)	56.6+/−10.8	58.4+/−9.5	0.0003 (t-test)

Reproducibility of the presence or absence of papillovascular abnormalities was assessed using the kappa statistics for the two graders, which were 0.70, 0.71 and 0.90 for presence of cilioretinal arteries, tilted discs and situs inversus, respectively, indicating substantial agreement.

**Fig. 1:**
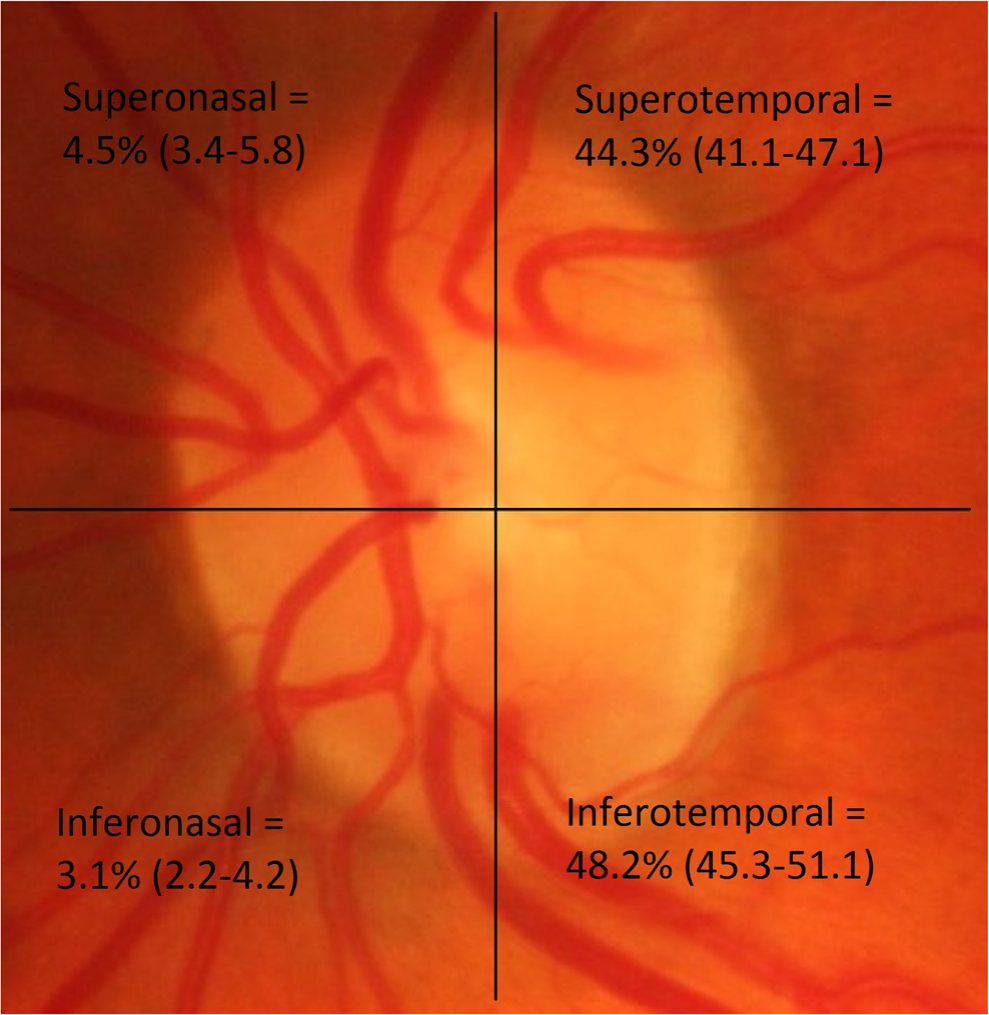
Locations of cilioretinal arteries by quadrant

**Fig. 2:**
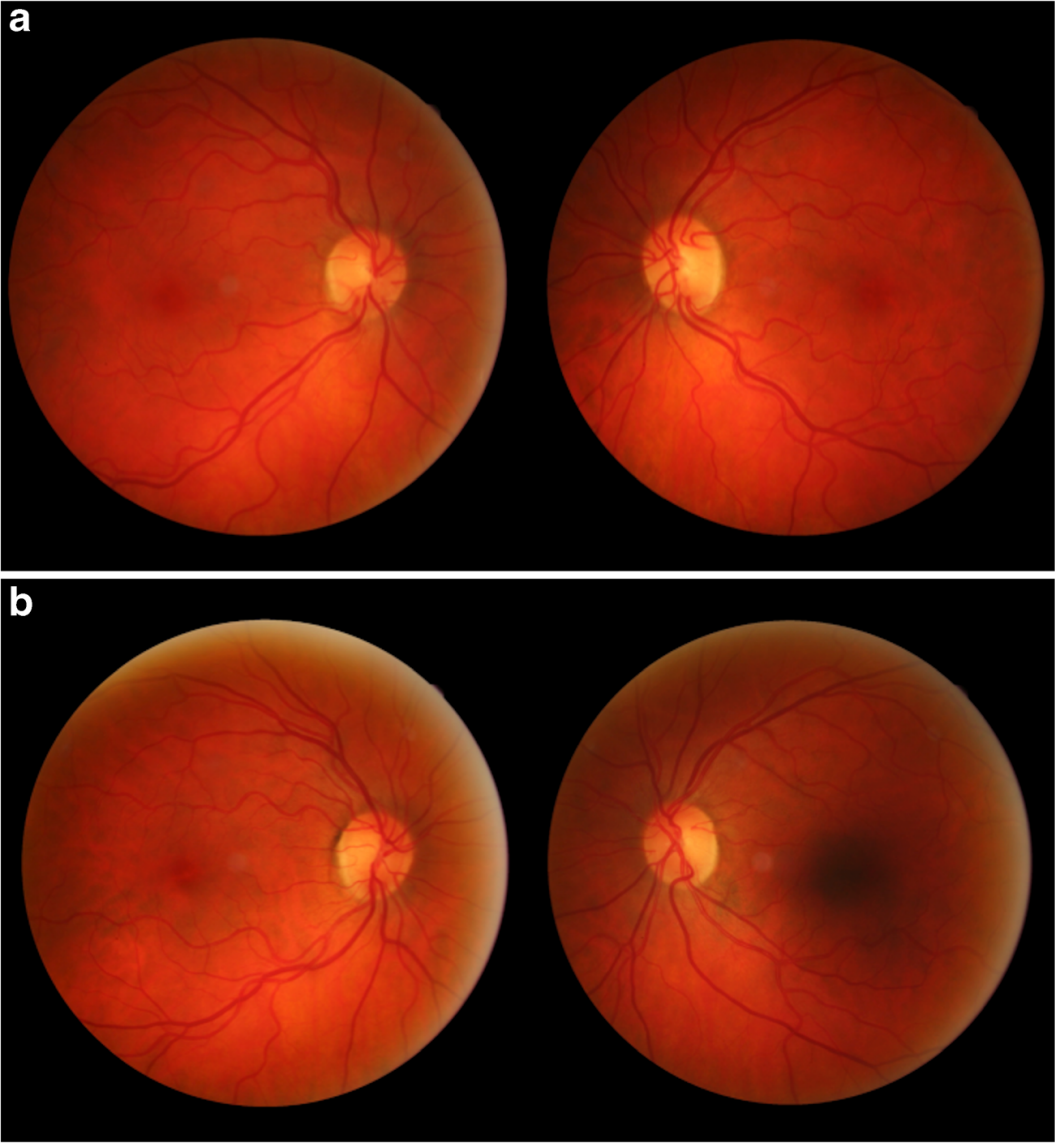
**a** Bilateral cilioretinal arteries (CRA) in a pair of monozygotic twins. Twin 1: right eye, one supero-temporal CRA and two infero-nasal CRA; left eye, one supero-temporal CRA **b** Bilateral cilioretinal arteries (CRA) in a pair of monozygotic twins. Twin 2: right eye, one infero-temporal CRA; left eye, one supero-temporal CRA, one infero-nasal CRA

**Fig. 3:**
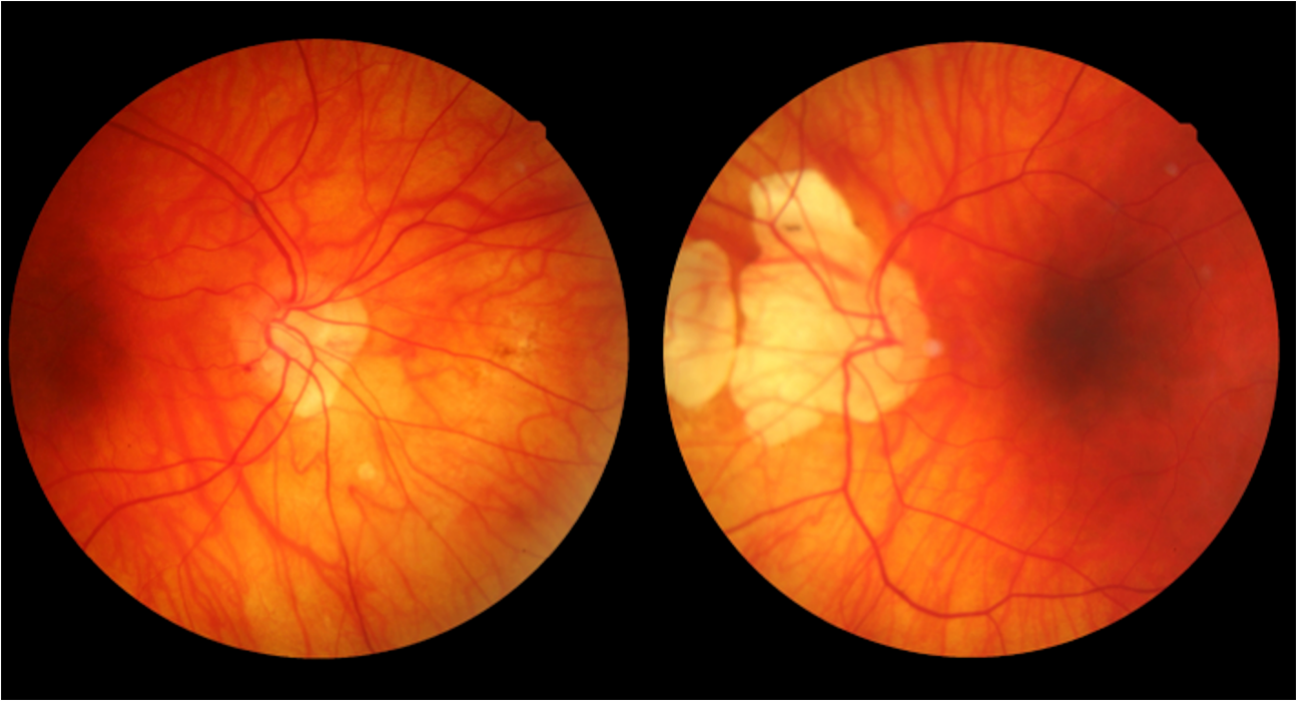
Bilateral situs inversus (discordant twin pair)

The prevalence of cilioretinal arteries was very similar in both eyes (Table 2a). The majority of these, 92.5% (95% CI = 90.9–93.9%), were located temporally (Fig. 1). 7.5% (6.1–9.2%) were located nasally. With respect to the horizontal meridian, 48.7% (45.9–51.6%) were located superiorly, and 51.3% (48.4–54.1%) inferiorly. 45.0% of individuals had a cilioretinal artery (Table 2). Probandwise concordance rates for cilioretinal arteries were consistently higher in MZ twin pairs, and this difference was significant when looking at rates for the left eye, either eye and both eyes. The heritability of having a cilioretinal artery (using the best fitting AE model, see supplementary Table S1) in either eye was 49.4% (38.1–59.7%), and in both eyes was 32.9% (10.4–53.3%) (Figs. 2 and 3).

**Table 2:** Prevalence, concordance and heritability of cilioretinal artery, tilted disc and situs inversus

Phenotype	Prevalence (95% CI)				Number of concordant pairs		Number of discordant pairs		Concordance (95% CI)		Heritability (95%CI)
	Overall	MZ twins	DZ twins	p-value	MZ	DZ	MZ	DZ	MZ pairs	DZ pairs	
Right eye CRA	476/1665 = 28.6% (26.5–30.8)	272/1014 = 26.8% (24.0–29.7)	204/651 = 31.4% (29.3–36.9)	0.05	45	27	144	116	38% (34–43)	32% (26–37)	25.2% (10.6–39.3)
Left eye CRA	446/1634 = 27.3% (25.2–29.5)	263/989 = 26.6% (23.8–29.4)	183/645 = 28.4% (24.9–32.1)	0.42	40	19	151	115	35% (30–39)	25% (20–30)	18.2% (3.3–33.1)
CRA in at least one eye	689/1530 = 45.0% (42.6–47.5)	389/887 = 43.9% (40.5–47.2)	300/643 = 46.7% (42.6–50.9)	0.27	113	58	152	140	60% (55–64)	45% (39–51)	49.4% (38.1–59.7)
CRA in both eyes	168/1530 = 11.0% (9.5–12.7)	90/887 = 10.1% (8.1–12.1)	78/643 = 12.1% (9.4–14.9)	0.23	12	2	63	63	28% (23–32)	6% (3–8)	32.9% (10.4–53.3)
Right eye tilted disc	6/1665 = 0.4% (0.2–0.8)	4/1014 = 0.4% (0.0–0.9)	2/651 = 0.3% (0.0–0.8)	0.78	1	0	2	2	–	–	–
Left eye tilted disc	22/1634 = 1.4% (0.9–2.0)	16/989 = 1.6% (0.8–2.4)	6/645 = 1.0% (0.2–1.8)	0.31	3	1	5	2	–	–	–
Tilted disc in at least one eye	18/1530 = 1.2% (0.8–1.9)	12/887 = 1.4% (0.6–2.2)	6/643 = 0.9% (0.1–1.7)	0.41	3	1	6	4	–	–	–
Tilted disc in both eyes	4/1530 = 0.3% (0.1–0.7)	3/887 = 0.3% (0.0–0.7)	1/643 = 0.1% (0.0–0.5)	0.56	1	0	1	1	–	–	–
Right eye situs inversus	5/1665 = 0.3% (0.1–0.7)	3/1014 = 0.3% (0.0–0.7)	2/651 = 0.3% (0.0–0.8)	0.96	1	0	1	2	–	–	–
Left eye situs inversus	12/1634 = 0.7% (0.4–1.3)	9/989 = 0.9% (0.3–1.5)	3/645 = 0.5% (0.1–1.1)	0.41	0	0	4	2	–	–	–
Situs inversus in at least one eye	8/1530 = 0.5% (0.3–1.0)	5/887 = 0.6% (0.1–1.1)	3/643 = 0.5% (0.1–1.2)	0.93	1	0	3	3	–	–	–
Situs inversus in both eyes	3/1530 = 0.2% (0.1–0.5)	2/887 = 0.2% (0.1–0.6%)	1/643 = 0.2% (0.0–0.5)	0.84	0	0	2	1	–	–	–

Abbreviations: *CRA* clioretinal artery, *MZ* monozygotic, *DZ* dizygotic

Situs inversus was present in 0.5% (0.3–1.0%) of subjects, and tilted discs in 1.2% (0.8–1.9%), (Table 2c and b). We were unable to meaningfully calculate heritabilities or probandwise concordance rates for situs inversus and tilted discs because the number of subjects with these phenotypes was too low.

We did not find any significant difference between cilioretinal artery prevalence based on birthweight (Table 3). Prevalence in the extremely low birthweight group (<1000 g) was 50.0, compared to 43.1 in the normal birthweight (2500-4200 g) group (*p* = 0.727).

**Table 3:** Cilioretinal artery prevalence by birth weight

Birthweight	Prevalence of cilioretinal artery in either eye (95% CI)	p-value, (compared to normal birthweight)
Extremely low birthweight (<1000 g)	50.0 (14.7–85.3)	0.727
Very low birthweight (1000 g - 1500 g)	45.2 (30.9–60.5)	0.787
Low birthweight (1500 g - 2500 g)	47.7 (39.7–55.7)	0.322
Normal birthweight (2500-4200 g)	43.1 (39.1–47.2)	–
p trend	*p* = 0.398	

## Discussion

Our study found a cilioretinal artery prevalence of 28.6% and 27.3% in right and left eyes; 45.0% of participants had at least one cilioretinal artery, and 11.0% had bilateral cilioretinal arteries. The heritability for having a cilioretinal artery in both eyes was 32.9% (10.5–53.3%), and in either eye was 49.4% (38.1–59.7%), suggesting that there is a genetic element in determining the presence of a cilioretinal artery, but environmental factors explain the greater residual variance. Our twin study found no evidence of a shared or common environmental (C) effect, although twin studies are underpowered to detect these effects compared to other family-based models.

There are disease processes that suggest a role for environmental and genetic influences on vasculature development, such as retinopathy of prematurity (environmental), and inherited familial exudative vitreoretinopathy (genetic).

Previous studies have examined factors influencing retinal blood vessel geography [9, 23]. In a group of 47 women those who were born preterm at a median of 30 weeks had a significantly different pattern of retinal vasculature to women born at full term [23]. When 15 children with birth weights of ≤2500 g were compared to 370 children with normal birth weights the prevalence of cilioretinal arteries in the lower birth weight children was 53%, compared to 27.3% in the normal birth weight group (*p* < 0.05) [9]. Low birth weight has also been associated with structural and functional changes in the vascular tree throughout the body [24]. Hypoxia, maternal nutrition, and maternal glucocorticoid levels are other factors that may affect fetal levels of VEGF and blood vessel development [25]. However, our study found no significant link between cilioretinal artery prevalence and birth weight (Table 3).

Our study has built upon the previous study by Taarnhoj et al. by examining a much larger twin sample (862 complete twin pairs, as compared to 112). Our heritability for the presence of a cilioretinal artery in both eyes, using the best fitting AE model, was 32.9% (CI 10.4–53.3%). Conversely, Taarnhoj et al. found a considerably higher heritability than our study: heritability for both eyes using the best fitting AE model was 74% (95% CI 0.34–0.94). Despite the difference between our results, our confidence intervals overlap, and it may be that the heritability is not significantly different between the two studies.

The differing heritability point estimates could be due to different populations, methods, or chance. The mean age in the Danish study was 35 years, whereas in this study it was 57 years, and there could be a cohort effect. The methods used for identifying cilioretinal arteries were similar, but not identical: both used fundus photographs, (although the Danish images were red-free and TwinsUK were color). Taarnhoj et al. had a higher proportion of male subjects (44% as compared to 1%). If the relevant genes were carried on the X-chromosome, then it could be more heritable in male twin pairs. Differences in the health or environment of the populations or the maternal populations could also have caused different inheritance patterns. However, it is hard to know whether any of these differences could have influenced the heritability of cilioretinal arteries, particularly as the prevalence was similar in both studies, with a per-eye prevalence of 28.8% and a per person prevalence of 45.1% in the Danish twins and 27.9% and 45.0% in the UK twins. These figures are similar to those found in a study of subjects of European origin that used fluorescein angiography (prevalence 49.5%) [4]. Fewer (35%), had a cilioretinal artery in a Chinese study [3].

Situs inversus was found in 0.52% of eyes and 0.5% of individuals. Unfortunately, there were too few affected subjects to calculate meaningful heritability estimates. The only previous study we have identified found 0.21% of 4324 Korean subjects attending a glaucoma clinic in Seoul had situs inversus of the optic disc [19].The different ethnicity of this group and attendance at a glaucoma clinic makes it difficult to compare with our group of healthy Caucasians.

Tilted discs were found in 0.8% of eyes and 1.2% of individuals; again with insufficient affected twin pairs for accurate calculation of heritability. The only original study we identified which examined the prevalence of tilted optic discs found a similar figure of 1.6% [12]. The tilted disc syndrome (as opposed to a tilted disc in isolation), has been reported in three successive generations of a family with variable degrees of expression, which might indicate dominant inheritance [18]. It is important to note our study is of tilted discs, rather than the tilted disc syndrome, which consists of a number of signs including superotemporal visual field loss. Some of our subjects may have had tilted discs secondary to myopia, but excluding myopes would have resulted in even fewer cases.

Limitations to our study include the fact that it examines a predominantly female, Caucasian twin population and therefore may not be representative of other population groups. We graded color fundus photographs only, and the most accurate method of identifying a cilioretinal artery is by using fluorescein angiography, because the artery will fill with dye earlier than the rest of the retinal arterial circulation, although as stated the prevalence we obtained was similar to other studies [4, 5]. Images were viewed roughly in sequence of twin pairs, so it is possible that some bias occurred. However the grader did not know zygosity, so heritability calculations should not have been affected. Some images could have been graded incorrectly. This would likely increase the estimate for “E” (unique environment, which includes measurement error), and therefore reduce the estimate for heritability.

This study suggests that cilioretinal arteries are moderately heritable; individual environmental factors explain a considerable proportion of the variance, and it may be that stochastic events contribute to their development. Other factors contributing to the formation of cilioretinal arteries remain unknown; we found no association with birth weight. Further well-studied birth cohorts with information on maternal and in utero factors might offer further insights, and these heritability data suggest that genetic studies might allow additional understanding of the etiology of congenital papillovascular variants.

## Electronic supplementary material

ESM 1 (DOCX 17 kb)
