# Supplementary material for: A twin study of cilioretinal arteries, tilted discs and situs inversus
Source: Graefes Arch Clin Exp Ophthalmol. 2017 Dec 14;256(2):333–40. doi: 10.1007/s00417-017-3859-7 (PMC5790863; doi:10.1007/s00417-017-3859-7)
Supplement: Supplementary file 1 — (DOCX 17 kb) [file 417_2017_3859_MOESM1_ESM.docx]

**Supplementary table/Appendix A1. Results of the model fitting for all variables** For each model the minus 2 log-likelihood (minus2ll), degrees of freedom (df), Akaike information criertion (AIC), difference in log-likelihood (difLL), difference in degrees of freedom (diffdf) and p value for model comparison are given. AIC is used to evaluate the most parsimonious model and the best fitting model is highlighted in bold. A = additive genetic effects, D = dominant genetic effects, C = common environmental effects and E = unique environmental effects (and measurement error)

| **Variable** | **Model** | **Comparison** | **minus2LL** | **df** | **AIC** | **diffLL** | **diffdf** | **p** |
| --- | --- | --- | --- | --- | --- | --- | --- | --- |
| **Cilioretinal artery right eye** | ACE | - | 1918.93 | 1607.00 | -1295.07 | NA | NA | NA |
|  | **AE** | **ACE** | **1918.93** | **1608.00** | **-1297.07** | **0.00** | **1** | **1** |
|  | CE | ACE | 1920.50 | 1608.00 | -1295.50 | 1.57 | 1 | 0.21 |
|  | E | ACE | 1930.08 | 1609.00 | -1287.92 | 11.15 | 2 | 0.00 |
| **Cilioretinal artery left eye** | ACE | - | 1898.37 | 1613.00 | -1327.63 | NA | NA | NA |
|  | **AE** | **ACE** | **1898.37** | **1614.00** | **-1329.63** | **0.00** | **1** | **1.00** |
|  | CE | ACE | 1900.77 | 1614.00 | -1327.23 | 2.40 | 1 | 0.12 |
|  | E | ACE | 1904.06 | 1615.00 | -1325.94 | 5.69 | 2 | 0.06 |
| **Cilioretinal artery either eye** | ACE | - | 2227.59 | 1723.00 | -1218.41 | NA | NA | NA |
|  | **AE** | **ACE** | **2227.59** | **1724.00** | **-1220.41** | **0.00** | **1** | **1.00** |
|  | CE | ACE | 2236.46 | 1724.00 | -1211.54 | 8.87 | 1 | 0.00 |
|  | E | ACE | 2289.51 | 1725.00 | -1160.49 | 61.92 | 2 | 0.00 |
| **Cilioretinal artery both eyes** | ACE | - | 1040.17 | 1535.00 | -2029.83 | NA | NA | NA |
|  | **AE** | **ACE** | **1040.17** | **1536.00** | **-2031.83** | **0.00** | **1** | **1.00** |
|  | CE | ACE | 1044.16 | 1536.00 | -2027.84 | 3.99 | 1 | 0.05 |
|  | E | ACE | 1048.29 | 1537.00 | -2025.71 | 8.12 | 2 | 0.02 |
